# Fluid shear stress enhances T cell activation through Piezo1

**DOI:** 10.1186/s12915-022-01266-7

**Published:** 2022-03-09

**Authors:** Jacob M. Hope, Jenna A. Dombroski, Rebecca S. Pereles, Maria Lopez-Cavestany, Joshua D. Greenlee, Samantha C. Schwager, Cynthia A. Reinhart-King, Michael R. King

**Affiliations:** Department of Biomedical Engineering, Vanderbilt University, 5824 Stevenson Center, Nashville, TN 37235 USA

**Keywords:** Fluid shear stress, Piezo1, T cells, Mechanotransduction

## Abstract

**Background:**

T cell activation is a mechanical process as much as it is a biochemical process. In this study, we used a cone-and-plate viscometer system to treat Jurkat and primary human T cells with fluid shear stress (FSS) to enhance the activation of the T cells through mechanical means.

**Results:**

The FSS treatment of T cells in combination with soluble and bead-bound CD3/CD28 antibodies increased the activation of signaling proteins essential for T cell activation, such as zeta-chain-associated protein kinase-70 (ZAP70), nuclear factor of activated T cells (NFAT), nuclear factor kappa B (NF-κB), and AP-1 (activator protein 1). The FSS treatment also enhanced the expression of the cytokines tumor necrosis factor alpha (TNF-α), interleukin 2 (IL-2), and interferon gamma (IFN-γ), which are necessary for sustained T cell activation and function. The enhanced activation of T cells by FSS was calcium dependent. The calcium signaling was controlled by the mechanosensitive ion channel Piezo1, as GsMTx-4 and Piezo1 knockout reduced ZAP70 phosphorylation by FSS.

**Conclusions:**

These results demonstrate an intriguing new dynamic to T cell activation, as the circulatory system consists of different magnitudes of FSS and could have a proinflammatory role in T cell function. The results also identify a potential pathophysiological relationship between T cell activation and FSS, as hypertension is a disease characterized by abnormal blood flow and is correlated with multiple autoimmune diseases.

**Supplementary Information:**

The online version contains supplementary material available at 10.1186/s12915-022-01266-7.

## Background

In vivo, optimal T cell activation is both a mechanical and biochemical process [1, 2]. For example, the knockdown of the mechanotransductive protein Piezo1 was recently shown to reduce the expansion of T cells activated by mouse dendritic cells [3]. Piezo1 is a mechanosensitive ion channel that opens in response to physical forces, such as fluid shear stress (FSS), and allows for calcium influx [4, 5]. Calcium influx causes Piezo1 to transduce physical stimuli into biochemical responses, since calcium is a second messenger involved in multiple signaling pathways. One of these pathways is T cell activation, as calcium influx increases the activation of the transcription factors nuclear factor of activated T cells (NFAT), nuclear factor kappa B (NF-κB), and activator protein 1 (AP-1) [6–10]. These transcription factors then induce the production of cytokines important in sustained T cell activation, differentiation, and cytotoxicity [11, 12]. A previous study showed that fluid shear stress (FSS) induces calcium influx in single T cells through the use of a micropipette apparatus, suggesting that FSS through Piezo1 activation may be proinflammatory [13].

In this study, we treated Jurkat and primary human T cells with FSS using cone-and-plate viscometers. Cone-and-plate viscometers were used because the geometry of the cone-and-plate viscometer exposes each cell in the fluid to the same FSS regardless of the cell’s location [14]. Treating T cells with FSS in this controlled setting allowed for the investigation of the effect of FSS in T cell activation in physiological and pathophysiological contexts. Here, the effect of physiological FSS was investigated by treating the T cells with magnitudes of 0.5 to 5.0 dyn/cm^2^ of FSS [15]. However, in a pathophysiological context, hypertension is a disease in which blood flow is altered, is associated with abnormal cytokine levels, and is correlated with multiple autoimmune disorders [16, 17]. In certain regions of the vascular network of hypertensive patients, blood flow velocity is reduced, reducing FSS. In other areas, blood flow velocity is increased, raising FSS [18]. Understanding the relationship between FSS and altered T cell activation could identify new therapeutic targets for the treatment of autoimmune disorders in hypertensive patients.

Additionally, ex vivo T cell activation is an indispensable step for producing adoptive T cell immunotherapies [19]. Treating primary human T cells with FSS ex vivo using tools such as a cone-and-plate viscometer could be used to potentially improve the production of adoptive T cell immunotherapies.

To our knowledge, this study is the first to determine that FSS enhances T cell activation. Short FSS treatments of 1 h in combination with soluble and bead-bound CD3/CD28 antibodies enhanced the activation of important signaling proteins in T cell activation and boosted the expression of cytokines important in sustained T cell function. The enhanced activation of T cells was found to rely on calcium and activation of the mechanosensitive ion channel Piezo1. These observations suggest that Piezo1 and other mechanosensitive ion channels may be therapeutic targets for autoimmune diseases, or for improving adoptive T cell immunotherapies.

## Results

### FSS enhances ZAP70 phosphorylation in Jurkat cells treated with CD3/CD28 antibodies

**Fig. 1:**
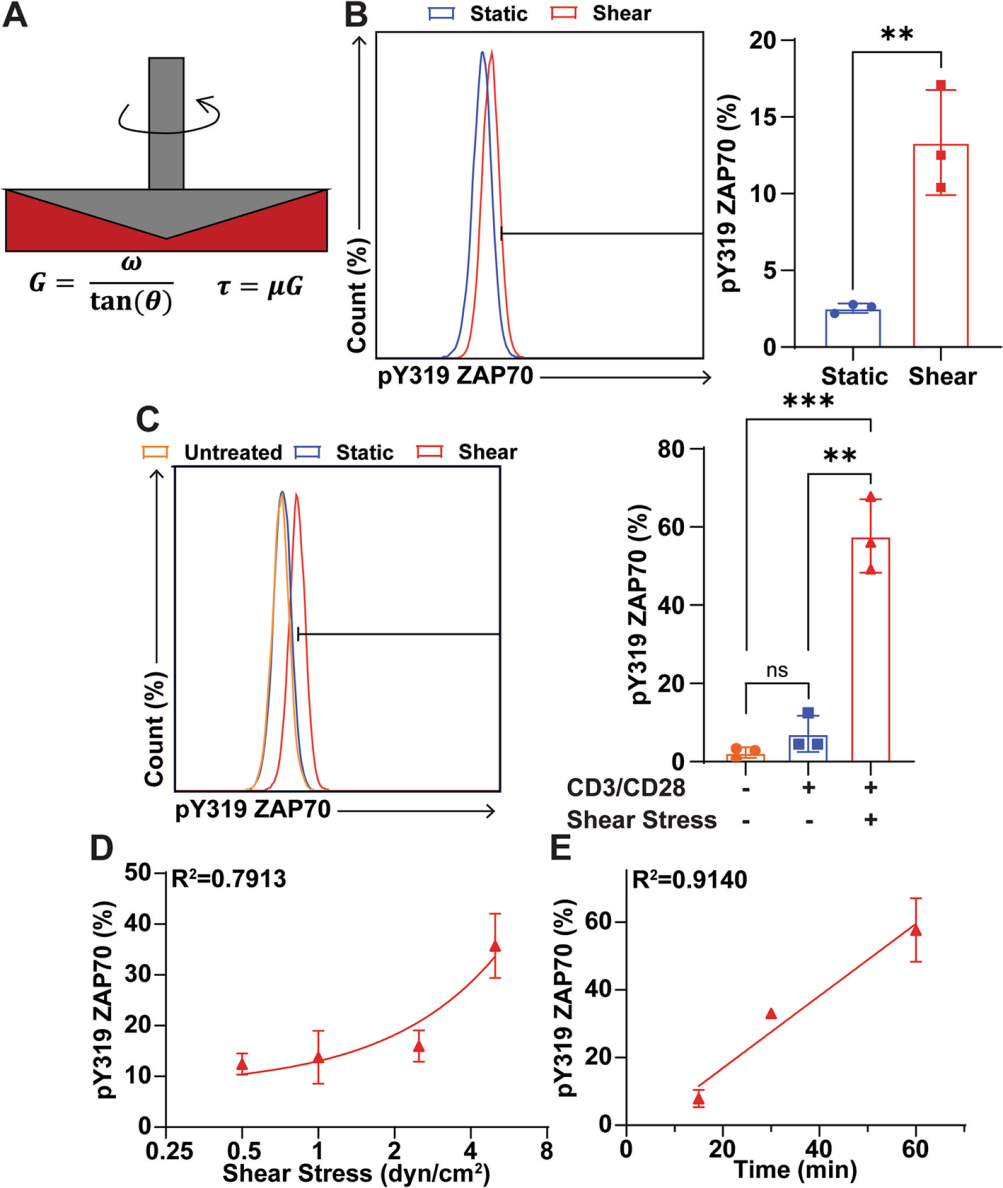
Phosphorylation of ZAP70 by CD3/CD28 antibody and FSS treatment. **A** Schematic of FSS treatment via cone-and-plate viscometer. *G* = shear rate, *ω* = angular velocity, *θ* = angle of cone, *τ* = fluid shear stress, *μ* = viscosity. **B** Representative flow plot and graph of ZAP70 phosphorylation of Jurkat cells treated with or without FSS (*N*=3). **C** Representative flow plot and graph of ZAP70 phosphorylation in Jurkat cells treated with or without FSS and CD3/CD28 antibodies (*N*=3). Untreated: Jurkat cells that were treated with neither FSS nor antibodies; static: Jurkat cells treated with only antibodies; shear: cells treated with antibodies and FSS. **D** ZAP70 phosphorylation of Jurkat cells treated with a constant concentration of CD3/CD28 antibodies for 1 h as a function of FSS magnitude (*N*=3). **E** ZAP70 phosphorylation of Jurkat cells treated with a constant concentration of CD3/CD28 antibodies and a constant magnitude of FSS as a function of time (*N*=3). Unpaired *t* tests were used to measure statistical significance between treatment groups. * *p*<0.05, ** *p*<0.01, *** *p*<0.005. Error bars are SD. Linear regression was used to measure correlation and determine if function slopes differed significantly from zero in **D** and **E**

The immortalized Jurkat cell line was used to model T cell activation by CD3/CD28 antibodies in combination with FSS in vitro. Jurkat cells were incubated with soluble CD3/CD28 antibodies with or without FSS for 1 h. Cone-and-plate viscometers were used to expose the cells to 5.0 dyn/cm^2^ FSS (Fig. 1A). However, FSS at sufficient magnitude has previously been shown to be cytotoxic to cells [20]. Levels of shear stress as high as 1000 dyn/cm^2^ are experienced at bifurcations in the heart and can damage the structure of cells [21]. Throughout FSS experiments, we maintained shear stress values at venous and arterial levels, which are 0.5–4.0 dyn/cm^2^ and 4.0-30.0 dyn/cm^2^, respectively [15]. The 1 h time point has been established in previous protocols as non-cytotoxic, although periods of fluid shear stress have been tested for as long as 2 h [22]. To determine if the level of FSS exposure was cytotoxic to the cells, an annexin V assay was done to measure cell viability. 1 h of FSS treatment at 5.0 dyn/cm^2^ did not significantly reduce cell viability (Additional file 1 Fig. S1). Jurkat cells were first treated with FSS without antibodies to determine the activating effect of FSS alone. This was quantified by measuring zeta-chain-associated protein kinase-70 (ZAP70) phosphorylation, which is phosphorylated following the activation of CD3 and is a necessary step for T cell activation [23]. FSS alone caused only a 10% increase in ZAP70 phosphorylation compared to the static control (Fig. 1B). When CD3/CD28 antibodies were combined with the FSS treatment, ZAP70 phosphorylation was significantly and dramatically increased in comparison to antibody only or the untreated controls. The increase in ZAP70 phosphorylation was about 50% greater when FSS was used with antibodies, compared to the ZAP70 phosphorylation of T cells treated with antibodies alone (Fig. 1C). Jurkat cells were then treated with varying amounts of FSS to determine the “dose response” of T cell activation to shear stress. A positive correlation of increasing ZAP70 phosphorylation with increasing amounts of FSS was observed, with *R*^2^=0.7913. Additionally, the slope of the best fit line was significantly different from zero (Fig. 1D). The dependence of Jurkat ZAP70 phosphorylation on FSS-antibody treatment time was also measured. There was a significant increase in ZAP70 phosphorylation with increasing FSS treatment times, with *R*^2^=0.9140. The best fit line between treatment time and ZAP70 phosphorylation was significantly different from zero (Fig. 1E).

### FSS in combination with CD3/CD28 antibodies increases the expression of later stage T cell activation markers in Jurkat cells

**Fig. 2:**
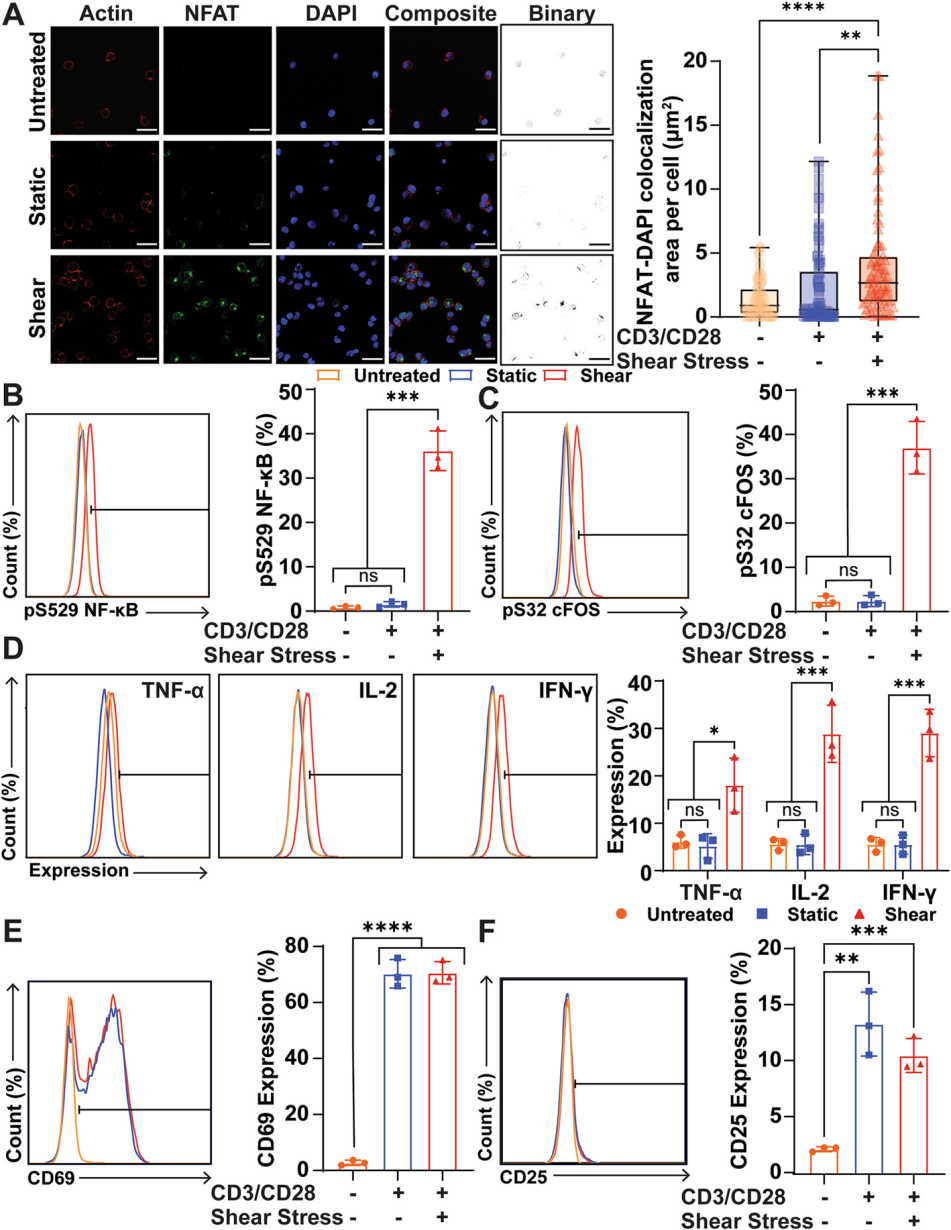
FSS increases later-stage markers of T cell activation in Jurkat cells. **A** Representative images of confocal images of Jurkat cells treated with combinations of FSS and CD3/CD28 antibodies. Area of colocalization between NFAT and DAPI was used to measure NFAT activation (untreated sample *n*=81, antibody-only sample *n*=81, antibody-FSS sample *n*=86, *N*=3, scale bars = 30 μm). Images were acquired on Zeiss 800 LSM with a 40x/1.1NA water immersion objective. **B** Representative flow plot and graph of NF-κB phosphorylation at serine 529 of Jurkat cells treated 1 h with antibodies and FSS (*N*=3). **C** Representative flow plot and graph of cFOS phosphorylation at serine 32 of Jurkat cells treated with antibodies and FSS as a measure of AP-1 activation after 1 h of treatment (*N*=3). **D** Flow plots and graph of TNF-α, IL-2, and IFN-γ expression 24 h after FSS stimulation and antibody treatment (*N*=3). **E** Representative flow plot and graph of CD69 expression 24 h after FSS and antibody treatment (*N*=3). **F** Representative flow plot and graph of CD25 expression 24 h after FSS and antibody treatment (*N*=3). Unpaired *t* tests were used to measure statistical significance between treatment groups. * *p*<0.05, ** *p*<0.01, *** *p*<0.005, **** *p*<0.001. Error bars are SD

For proper T cell activation, the transcription factors NFAT, NF-κB, and AP-1 need to be activated for the transcription of important proteins and cytokines, such as tumor necrotic factor alpha (TNF-α), interleukin 2 (IL-2), and interferon gamma (IFN-γ) [24]. The activation of each transcription factor was measured after 1 h of stimulation with FSS and CD3/CD28 antibodies. NFAT activation of Jurkat cells was measured by quantifying the colocalization of NFAT with the nucleus using confocal microscopy, since only the active conformation of NFAT reveals a nuclear localization signal [25]. The colocalization was measured via antibody staining for NFAT and DAPI staining of the nucleus. When the Jurkat cells were treated with CD3/CD28 antibodies and FSS, a significant increase in NFAT-nucleus colocalization was observed relative to untreated, or antibody-only treated Jurkat cells (Fig. 2A). NF-κB activation was quantified by measuring the phosphorylation of NF-κB using flow cytometry, as NF-κB phosphorylation is indicative of its activation [26]. AP-1 activation was quantified by measuring the phosphorylation of cFOS, since cFOS must be phosphorylated to form the AP-1 complex [27]. FSS in combination with CD3/CD28 antibodies significantly increased the activation of NF-κB and AP-1 compared to untreated and antibody-only treated Jurkat cells (Fig. 2B, C).

The expression of the cytokines TNF-α, IL-2, and IFN-γ was measured 24 h after 1 h of FSS treatment, to determine if the increased activation of the three transcription factors correlated with increased expression of proteins necessary for sustained T cell function and activation. The expression of each cytokine was significantly increased when Jurkat cells were treated with FSS for 1 h compared to both control groups (Fig. 2D). The expression of the activation markers CD69 and CD25 were also measured 24 h after FSS treatment. Both the FSS-antibody treated group and the antibody-only treated group showed a significant increase in CD69 and CD25 expression compared to the untreated control. There was no significant difference in CD69 and CD25 expression between either treatment group (Fig. 2E, F).

### CD3/CD28 antibody-coated beads with FSS increase activation of Jurkat cells

**Fig. 3:**
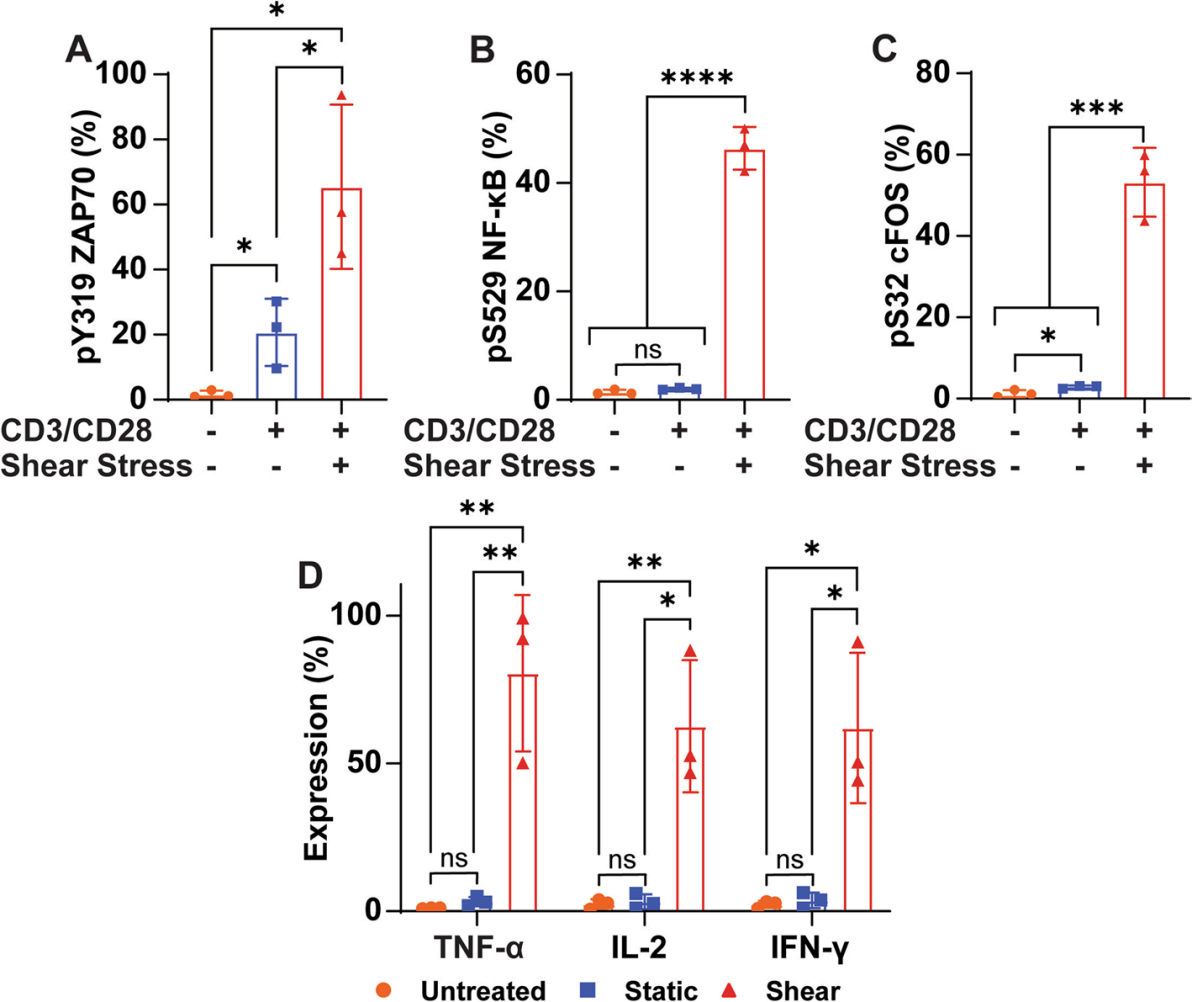
FSS increases activation by CD3/CD28 coated beads. **A** Graph of increased ZAP70 phosphorylation in Jurkat cells treated with FSS and CD3/CD28 antibody beads for 1 h compared to CD3/CD28 beads alone and untreated cells (*N*=3). **B** Graph of NF-κB phosphorylation for Jurkat cells activated with or without FSS, and with or without beads coated with CD3/CD28 for 1 h (*N*=3). **C** Graph of cFOS phosphorylation after Jurkat cells were treated with combinations of CD3/CD28 coated beads and FSS for 1 h (*N*=3). **D** Cytokine expression 24 h following 1 h of FSS treatment in combination with CD3/CD28 coated beads (*N*=3). Unpaired t tests were used to measure statistical significance between treatment groups. * *p*<0.05, ** *p*<0.01, *** *p*<0.005, **** *p*<0.001. Error bars are SD

FSS was also tested with CD3/CD28 antibody-coated beads to determine if FSS could also enhance the activation of Jurkat cells activated using beads. The Jurkat cells were stimulated with or without FSS for 1 h in combination with antibody-coated beads. Similar to soluble antibodies, the FSS treatment significantly increased the phosphorylation of ZAP70 compared to the bead only and the untreated control. The bead-only control also had a significant increase in ZAP70 phosphorylation compared to the untreated control, but it was less pronounced than the FSS treated group (Fig. 3A). FSS also significantly enhanced the phosphorylation of the transcription factors NF-κB and cFOS after 1 h of treatment with beads compared to the bead only and untreated controls. The bead-only control only significantly increased phosphorylation of cFOS, not NF-κB (Fig. 3B, C). Cytokine expression by the Jurkat cells was also significantly increased by FSS when the Jurkat cells were treated with CD3/CD28 coated beads. TNF-α, IL-2, and IFN-γ all were greatly upregulated in the FSS conditions compared to the control groups (Fig. 3D).

### FSS enhancement of T cell activation in Jurkat cells is Piezo1 and calcium dependent

**Fig. 4:**
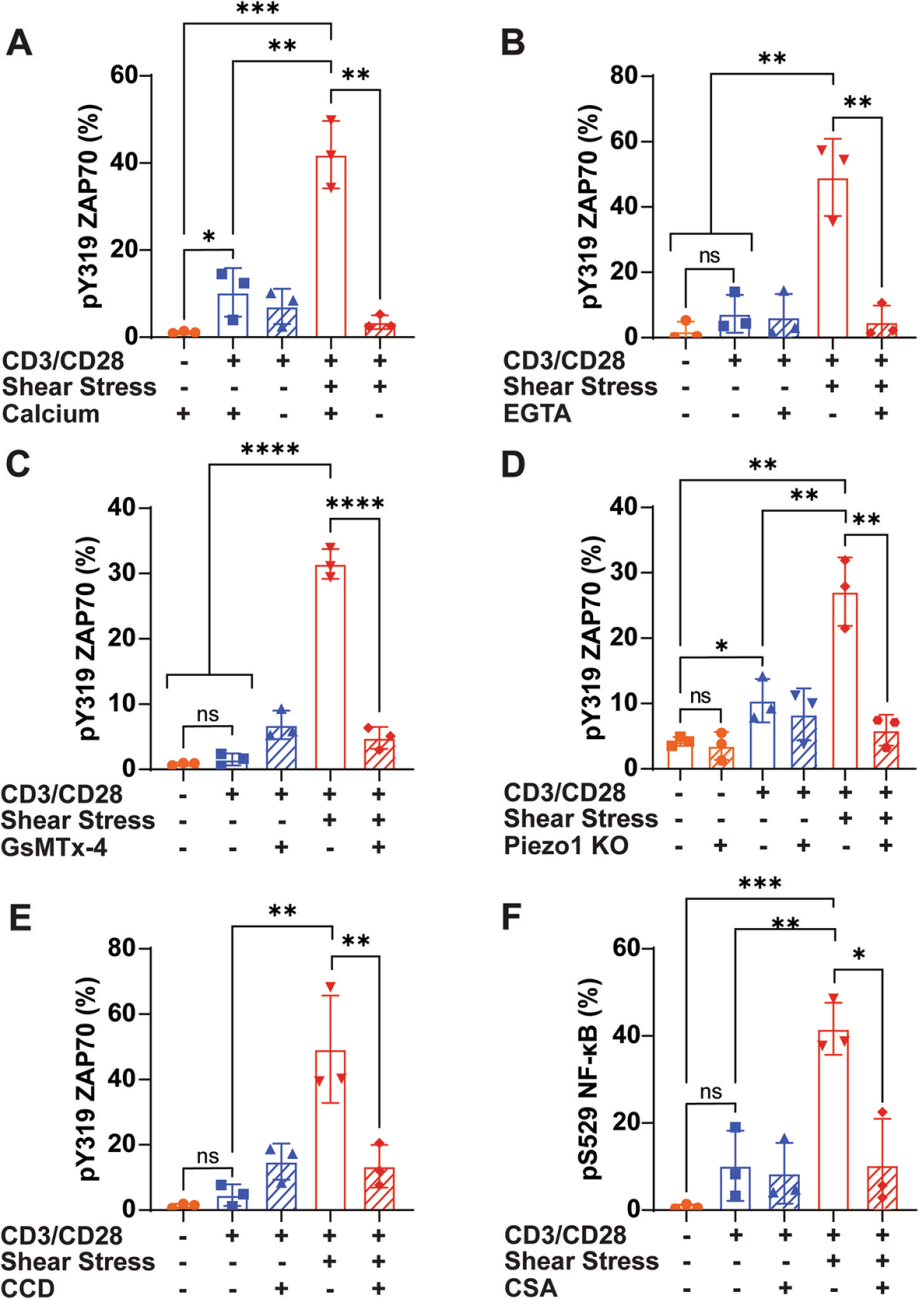
Calcium influx is essential for enhanced T cell activation by FSS in Jurkat cells. **A** ZAP70 phosphorylation of Jurkat cells treated with FSS and antibodies in calcium-free, or calcium-containing buffer (*N*=3). **B** ZAP70 phosphorylation of Jurkat cells treated with FSS and antibodies with or without the calcium chelator EGTA (*N*=3). **C** ZAP70 phosphorylation of Jurkat cells treated with FSS and antibodies with or without the mechanosensitive ion channel blocker GsMTx-4 (*N*=3). **D** ZAP70 phosphorylation of Cas9 control Jurkat cells and Piezo1 KO Jurkat cells treated with FSS and antibodies (*N*=3). **E** ZAP70 phosphorylation of Jurkat cells treated with FSS and antibodies pretreated with 10 μM CCD for 30 min (*N*=3). **F** NF-κB phosphorylation of Jurkat cells treated with FSS and antibodies pretreated with 5 μM CSA for 30 min (*N*=3). Unpaired *t* tests were used to measure statistical significance between treatment groups. * *p*<0.05, ** *p*<0.01, *** *p*<0.005, **** *p*<0.001. Error bars are SD

To determine if FSS-enhanced T cell activation acted through calcium influx, Jurkat cells were treated for 1 h with combinations of antibodies plus FSS in calcium-free, or calcium-containing HBSS buffer. Jurkat cells treated with FSS and antibodies in calcium-containing buffer showed a significant increase in ZAP70 phosphorylation compared to Jurkat cells treated with FSS and antibodies in calcium-free buffer. The Jurkat cells treated with FSS and antibodies in calcium-free buffer did not show a significant increase in ZAP70 phosphorylation compared to Jurkat cells treated with or without antibodies in calcium-free buffer (Fig. 4A). Next, Jurkat cells were pretreated with or without the calcium chelator ethylene glycol-bis(2-aminoethylether)-N,N,N′,N′-tetraacetic acid (EGTA) at a concentration of 2 mM for 30 min. EGTA inhibited the enhanced phosphorylation of ZAP70 that was previously observed in response to FSS and antibody treatment in Jurkat cells. Jurkat cells treated with EGTA, FSS, and antibodies showed a significant reduction in ZAP70 phosphorylation compared to Jurkat cells treated with FSS and antibodies only (Fig. 4B).

Piezo1 is a mechanosensitive ion channel that can be activated by FSS and is known to transport calcium when active. To determine if Piezo1 may play a role in the enhanced T cell activation by FSS, Jurkat cells were treated with 10 μM GsMTx-4 30 min prior to FSS and antibody treatment. GsMTx-4 is a relatively specific inhibitor of Piezo1, but is known to inhibit other mechanosensitive channels as well, such as TRPC6 [28]. GsMTx-4 caused a significant reduction in ZAP70 phosphorylation for Jurkat cells treated with FSS and antibodies in comparison to Jurkat cells treated with FSS, antibodies, and no GsMTx-4 (Fig. 4C). This indicates that FSS enhances T cell activation by inducing the activation of mechanosensitive ion channels that result in calcium influx. To more specifically quantify the degree to which Piezo1 is the ion channel responsible for the enhanced activation of Jurkat cells by FSS, Piezo1 was knocked out in Jurkat cells using CRISPR/Cas9 technology. The knockout (KO) of Piezo1 was confirmed via western blot (Additional file 2 Fig. S2). In Jurkat Piezo1 KO cells, FSS treatment did not increase ZAP70 phosphorylation compared to untreated, or antibody-only treated cells. However, in the Cas9 control Jurkat cells, the FSS treatment significantly increased ZAP70 phosphorylation compared to untreated and antibody-only treated cells. Additionally, ZAP70 phosphorylation was significantly greater in Cas9 control Jurkat cells compared to Jurkat Piezo1 KO cells when both were treated with FSS (Fig. 4D).

Downstream effects of calcium influx were also investigated to identify if they play a role in enhancing T cell activation in the presence of FSS. Actin polymerization has previously been identified as being necessary for efficient T cell activation and is regulated by calcium influx [3]. Jurkat cells were pretreated with 10 μM of Cytochalasin D (CCD) 30 min prior to FSS treatment to inhibit actin polymerization. CCD significantly reduced the increased ZAP70 phosphorylation associated with FSS-antibody treatment, while not significantly altering the activation of Jurkat cells under the antibody-only condition (Fig. 4E).

Calcineurin is a phosphatase that is activated downstream of calcium influx and has previously been associated with NF-κB activation [29]. To determine if calcineurin is activated by calcium influx to increase transcription factor activation, calcineurin was inhibited by pretreating the Jurkat cells with 5 μM Cyclosporin A (CSA) for 30 min. CSA treatment significantly reduced NF-κB phosphorylation in Jurkat cells treated with FSS and antibodies for 1 h compared to Jurkat cells treated with FSS without CSA, suggesting that FSS activates calcineurin to boost transcription factor activation (Fig. 4F).

### FSS enhances activation of primary T cells

**Fig. 5:**
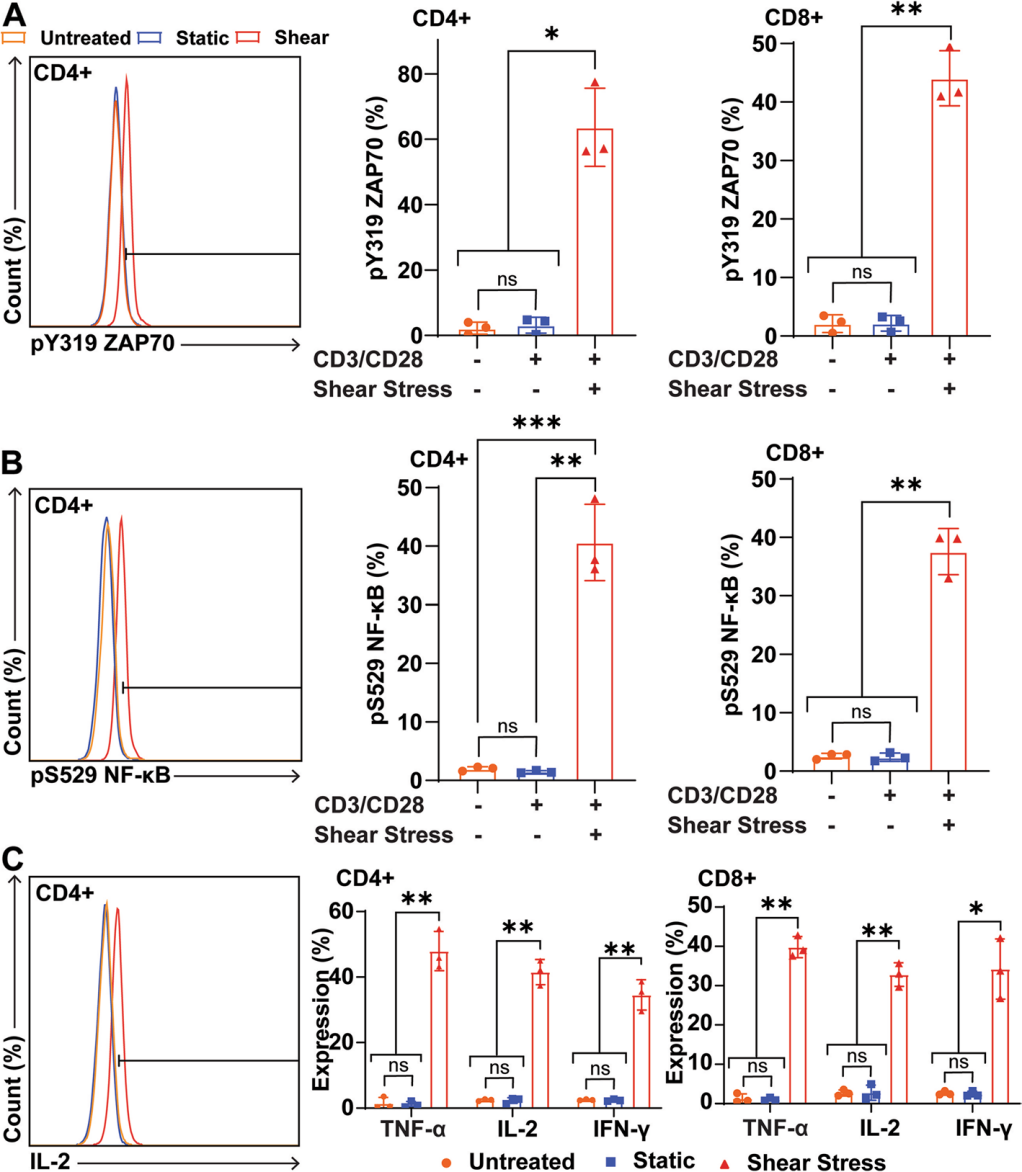
FSS enhances activation of primary human T cells. **A** Representative flow plot of ZAP70 phosphorylation of CD4-positive T cells. Graphs of ZAP70 phosphorylation for CD4- and CD8-positive T cells treated with FSS and antibodies (*N*=3). **B** Representative flow plot of NF-κB phosphorylation of CD4-positive T cells. Graphs of NF-κB phosphorylation for CD4- and CD8-positive T cells treated with FSS and antibodies (*N*=3). **C** Representative flow plot of IL-2 expression in CD4-positive T cells. Graphs of TNF-α, IL-2, and IFN-γ for CD4- and CD8-positive T cells treated with FSS and antibodies (*N*=3). Paired *t* tests were used to measure statistical significance between treatment groups. * *p*<0.05, ** *p*<0.01 *** *p*<0.005. Error bars are SD

For a more physiological model of T cell activation and to determine if the activating effects of FSS treatment are relevant to T cell therapies in the clinic, primary human T cells were treated with antibodies and FSS for 1 h. Primary T cells were isolated from the blood of 3 healthy donors using magnetic bead negative selection. Immediately following isolation, the T cells were treated with combinations of FSS and CD3/CD28 antibodies. The cells were stained for CD4 and CD8 to identify the helper and cytotoxic T cell subpopulations, respectively. The cells were also stained for various markers of T cell activation. FSS enhanced the phosphorylation of ZAP70 in both CD4- and CD8-positive T cell subpopulations compared to untreated and antibody-only control groups (Fig. 5A). This trend was also apparent with NF-κB phosphorylation where both the CD4- and CD8-positive T cells showed a significant increase in NF-κB phosphorylation compared to the two control groups (Fig. 5B). As mentioned above, cytokine expression is another essential step for T cell activation and function. Therefore, 24 h after 1 h of FSS treatment, the expression of TNF-α, IL-2, and IFN-γ was measured for primary T cells in both subpopulations. Again, the observations in Jurkat cells were recapitulated ex vivo with both primary T cell subpopulations. In CD4- and CD8-positive T cells there was a significant increase in all three cytokines when the T cells were treated with FSS and CD3/CD28 antibodies relative to untreated and antibody-only controls (Fig. 5C).

## Discussion

**Fig. 6:**
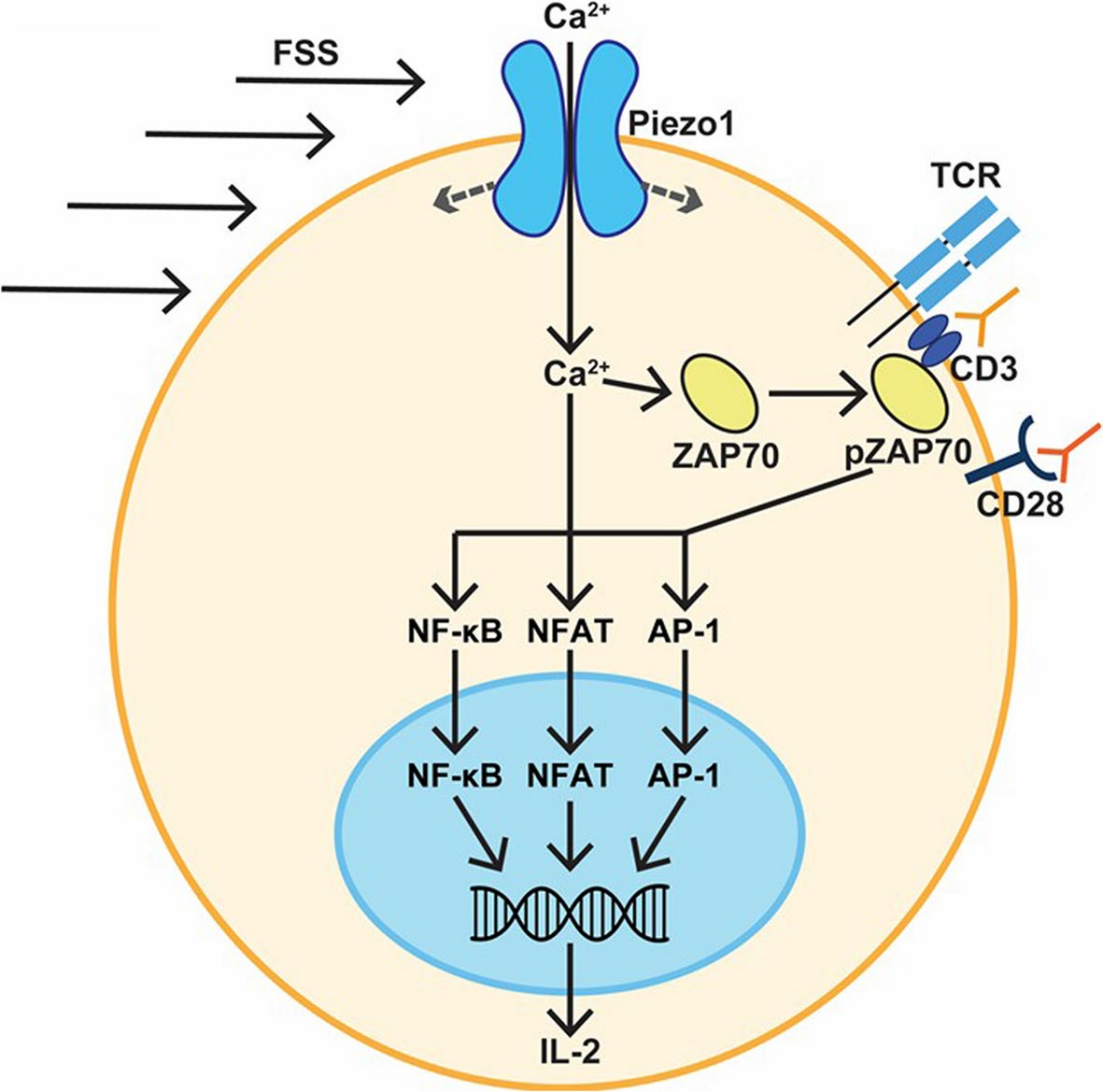
Schematic of enhanced T cell activation by FSS and CD3/CD28 antibodies. FSS activates Piezo1, enabling calcium influx. The calcium influx increases ZAP70 phosphorylation, and activation of the transcription factors NFAT, NF-κB, and AP-1. This in turn leads to increased expression of cytokines such as IL-2

To our knowledge, the present study is the first to demonstrate that FSS can enhance T cell activation (Fig. 6). FSS with soluble and bead bound CD3/CD28 antibodies significantly enhanced ZAP70 phosphorylation in immortalized Jurkat cells, and CD4- and CD8-positive human T cells (Figs. 1, 3, and 5). FSS was also found to increase the activation of the three major transcription factors associated with T cell activation in Jurkat cells (Fig. 2). These results are consistent with NF-κB in both subsets of primary T cells (Fig. 5). Finally, in both the Jurkat and primary T cells, upregulation of TNF-α, IL-2, and IFN-γ occurred when the cells were treated with FSS and activating antibodies (Figs. 2 and 4).

These results suggest that ex vivo FSS generated through devices, such as cone-and-plate viscometers, could potentially be used to enhance the activation of T cells in future T cell therapies. For instance, the significant increases of IFN-γ and IL-2 seen after FSS treatment could be beneficial because IFN-γ is known to induce apoptosis in cancer cells, and IL-2 is known to increase persistence of chimeric antigen receptor (CAR) T cells after reinfusion [30, 31]. However, in the current work, the T cells were only studied for a maximum of 24 h. If this method were to be used in T cell therapy production, it would be necessary to assay the T cells treated with FSS at later time points to measure how FSS affects their differentiation, as TNF-α, IL-2, and IFN-γ are known to play significant roles in these pathways [32–34]. Additionally, it would be of interest to measure how different FSS treatment regimens would affect T cell fate determination. More FSS magnitudes, durations, or oscillations could be tested to further optimize this protocol for enhanced T cell activation.

The mechanism of how FSS enhances T cell activation was also investigated (Fig. 6). It was found that the increase in ZAP70 phosphorylation was a calcium-mediated process, as removal of calcium either through chelation or a calcium-free buffer blocked the increased activation from FSS (Fig. 4). Additionally, events that are downstream of calcium signaling were inhibited to further establish the role of calcium influx in the FSS activation of T cells. CCD was used to block actin polymerization, which caused a significant reduction in ZAP70 phosphorylation. Calcineurin was inhibited via CSA and this resulted in reduced NF-κB phosphorylation, suggesting that calcium also works downstream of ZAP70 phosphorylation (Fig. 4). GsMTx-4 and CRISPR/Cas9 knockout of Piezo1 were used to specifically determine if the mechanosensitive ion channel Piezo1 was involved in this process, as a recent study identified that the chemical agonist of Piezo1 could be used to boost T cell activation [3]. Both methods of inhibition caused a decrease in ZAP70 phosphorylation in response to FSS treatment (Fig. 4). While our study identified Piezo1 as essential for FSS enhanced T cell activation, other mechanosensitive ion channels may also play roles in this process. Transient receptor potential cation channel subfamily V member 4 (TRPV4) activation has previously been shown to increase T cell activation through a calcium-mediated pathway, and TRPV4 is activated by FSS [35–37]. Additionally, Piezo1 has been implicated in having a pro-stimulatory effect on dendritic cells and macrophages, suggesting FSS could also have a pro-stimulatory effect in these cells as well [38, 39].

## Conclusions

This study demonstrated that the physiological FSS of 5.0 dyn/cm^2^ could enhance the activation of T cells when stimulated with antibodies against CD3/CD28 [15]. However, the results of our study could also relate to pathophysiological states, such as hypertension. We showed that increasing the magnitude of FSS further increased T cell activation as measured by ZAP70 phosphorylation. Hypertension is associated with increased blood flow velocity and therefore increased FSS, in certain regions of the body, such as in the left and right common carotid artery, and coronary artery [18, 40]. Hypertension also presents increased hazard ratios with multiple autoimmune disorders, such as type I diabetes, multiple sclerosis, and rheumatoid arthritis [41–43]. In each of these diseases, T cells are known to play a prominent role [44]. IFN-γ, which was upregulated by FSS in the present work, is known to further exacerbate autoimmune diseases, such as multiple sclerosis [45, 46]. Severe cytokine release syndrome by CAR T cell therapy and COVID-19 have also been shown to occur more frequently in hypertensive patients [47]. The results of this study and the correlation between hypertension and autoimmune diseases suggest that inhibition of Piezo1 and other mechanosensitive ion channels in T cells may hold therapeutic potential for autoimmune diseases and cytokine release syndromes.

## Methods

### Reagents and Antibodies

RPMI 1640 cell culture media, fetal bovine serum (FBS), and penicillin streptomycin were obtained from Invitrogen. Bovine serum albumin (BSA), dimethyl sulfoxide (DMSO), Triton, and CSA were obtained from Sigma. Hank’s balanced salt solution (HBSS) with calcium and magnesium and HBSS without calcium and magnesium were purchased from Gibco. GsMTx-4 was purchased from Abcam. CCD was obtained from Tocris. Ficoll-Paque was purchased from GE Healthcare. CRISPRMAX and Dynabeads Human T-Activator CD3/CD28 were purchased from ThermoFisher Scientific. Lamelli buffer was purchased from Bio-Rad. IRDye 800CW and IRDye 680RD were obtained from LI-COR. 32% Paraformaldehyde was purchased from Electron Microscopy Sciences. Anti-human functional CD3 (OKT3), functional CD28 (CD28.2), FITC-CD69 (FN50), APC-CD25 (CD25-4E3), FITC-TNF-α (MA11), PE-TNF-α (MAb11), FITC-IL-2 (MQ1-17H12), PE-IL-2 (MQ1-17H12), FITC-IFN-γ (4S.B3), PE-IFN-γ (4S.B3), PE-phospho-ZAP70 (n3kobu5), PE-phospho-cFos (cFosS32-BA9), PE-phospho-NF-κB (B33B4WP), APC-CD4 (RPA-T4), FITC-CD8 (RPA-T8), APC-CD3 (OKT3), APC-CD3-zeta (6B10.2), and PE-ZAP70 (1E7.2) were purchased from eBioscience. Anti-human Piezo1 (15939-1-AP) was purchased from Proteintech. Anti-human Glyceraldehyde-3-phosphate dehydrogenase (GAPDH) (MAB374) was purchased from EMD Millipore. Anti-human NFATc1 (7A6) was purchased from Biolegend. ActinRed 555 and DAPI were purchased from Molecular Probes. Annexin V and propidium iodide were obtained from BD Pharmingen.

### T cell activation by FSS

Jurkat or primary T cells were collected and resuspended at a concentration of 200,000 cells/mL in complete RPMI 1640 media. The cells were treated with or without functional grade antibodies targeting CD3 (OKT3) and CD28 (CD28.2) at a concentration of 2 μg/mL for both antibodies. Where indicated T cells were instead activated with Dynabeads Human T-Activator CD3/CD28 at a bead to cell ratio of 1:1. The T cells were then loaded into a Brookfield cone-and-plate viscometer for FSS treatment as described previously in Mitchell and King [48]. In a cone-and-plate viscometer the FSS magnitude is equal in all radial locations of the viscometer. The shear rate (*G*) is defined by the equation:

$$G=\frac{\omega }{\tan \left(\theta \right)}$$

where *ω* is the angular velocity (rad/s) and *θ* is the angle of the cone (rad). The flow field was assumed to be laminar, and the fluid assumed to be Newtonian. Therefore, the FSS (*τ*) is governed by the equation:

$$\tau =\mu G$$

where *μ* is the viscosity (cP) of the fluid. In these experiments, viscosity was approximately 2.5 cP. Before FSS treatment, the cone-and-plate viscometer was cleaned thoroughly with 70% ethanol. The stationary plate and the rotating cone were then incubated with 5% BSA for 1 h to block nonspecific adhesion. The cells were exposed to fluid shear stress ranging from 0.5 to 5.0 dyn/cm^2^ for times ranging from 15 to 60 min. Primarily, the cells were treated with FSS of magnitude 5.0 dyn/cm^2^ for 1 h.

Where indicated, Jurkat cells were pretreated with different compounds prior to antibody and FSS treatment. The Jurkat cells were incubated with either 5 μM Cyclosporin A (CSA), 10 μM Cytochalasin D (CCD), 10 μM GsMTx-4, or 2 mM EGTA 30 min prior to antibody and FSS treatment.

After FSS treatment, the cells were either stained immediately or cultured overnight in an incubator at 37 °C with 5% CO_2_. The cells stained for surface markers and cytokines were cultured overnight while the phospho staining was performed immediately.

### Cell culture

Jurkat cells were obtained from ATCC. Jurkat cells were cultured in RPMI 1640 supplemented with 10% FBS and 1% penicillin streptomycin in an incubator at 37 °C and 5% CO_2_.

### Primary T cell samples

Primary T cells were isolated from healthy human volunteers after informed consent under Vanderbilt University IRB Protocol #170222. On the day of activation, blood was collected in BD Vacutainer plastic blood collection tubes with sodium citrate. Peripheral blood mononucleocytes were purified from blood using ficoll gradient centrifugation. Following gradient centrifugation, T cells were isolated by using the Miltenyi Biotech human pan T cell isolation kit according to the manufacturer’s instructions. After incubation with the antibodies targeting all mononucleocytes except for T cells and magnetic beads in the kit, the mononucleocytes were perfused through a magnetic column. The flow through was collected to isolate the purified T cell population. Purified T cells were resuspended in RPMI 1640 supplemented with 10% FBS and 1% penicillin streptomycin. All experiments with human primary samples were approved by Vanderbilt University’s Institutional Review Board.

### Flow cytometry antibody staining

When staining for intracellular proteins, the cells were immediately fixed with 4% paraformaldehyde for 10 min. The cells were washed thoroughly and permeabilized with 100% ice cold methanol for 10 min. After washing the cells, the cells were stained with the indicated fluorescent-tagged antibodies for 15 min in 1% BSA. The fluorescence of each sample was measured using a Gauva easyCyte 5HT flow cytometer. Gating was performed using FlowJo software. For Jurkat samples, debris was gated out and fluorescence was quantified using FlowJo software. For primary T cell samples, after gating out cell debris, the CD4 and CD8 subpopulations were determined using fluorescence gating as shown in Additional file 3 Fig. S3.

For intracellular cytokine staining, the cells were allowed to incubate overnight following antibody and FSS treatment. 4 h prior to fixation, cells were treated with Golgiplug at 1:1000 ratio to block the secretion of cytokines. The cells were then stained using the same procedure as done for other intracellular proteins.

When staining for extracellular proteins such as CD69 and CD25, the cells were incubated with the indicated antibodies for 15 min in 1% BSA. The data collection and analysis were done following the same method as used for intracellular proteins.

### Confocal microscopy

Immediately after 1 h of FSS and antibody treatment, Jurkat cells were cytospun onto glass slides. The cells were fixed with 4% paraformaldehyde and permeabilized with 1% Triton. The cells were blocked with 5% BSA and stained overnight at 4 °C with antibodies against NFATc1. After staining for NFATc1 and washing, the cells were stained with an Alex Fluor 488 goat anti-rabbit secondary antibody, DAPI, and ActinRed 555 for 30 min. The cells were then imaged using a Zeiss LSM800 confocal microscope with a 40x/1.1NA water immersion objective. Image analysis was performed with ImageJ software. NFAT activation was measured using the fluorescent colocalization of the NFATc1 antibody and DAPI. The colocalization was quantified by calculating the area per cell where a cell was positive for both NFATc1 and DAPI fluorescence.

### Piezo1 knockout

CRISPR/Cas9 knockout was carried out using the Synthego Gene Knockout Kit V2 with sgRNAs targeting human Piezo1. The Jurkat cells were transfected for 3 days following the Synthego kit’s instructions for CRISPRMax-based knockout.

### Western Blot

Jurkat cells were lysed in Laemelli buffer. The lysates were resolved by SDS-PAGE electrophoresis and then transferred to PVDF membranes. The membranes were blocked with 5% BSA in TBS-Tween. The membranes were incubated overnight at 4 °C with primary antibodies against human Piezo1 and GAPDH. The membranes were then washed and stained with IRDye LI-COR fluorescent secondary antibodies. The membranes were imaged using an Odyssey CLx imager. The membrane fluorescence intensities were quantified using Image Studio Lite software. Uncropped blots can be found in Additional file 4.

### Annexin V

After Jurkat cells were treated with FSS for 1 h, the cells were washed with HBSS. The cells were then stained for 15 min with Annexin V and propidium iodide according to the manufacturer’s directions in HBSS with calcium and magnesium buffer. After staining, the cells were analyzed using flow cytometry. Cells negative for both Annexin V and propidium iodide were identified as being viable. Analysis was done in FlowJo.

### Statistics

Results are presented as means and standard deviation (SD). Statistical comparisons for Jurkat cells were done using unpaired Student’s *t* test. Statistical comparisons for primary T cells were done using paired *t* test. Linear regression was used to determine if the slopes significantly deviated from zero. Each experiment included at least 3 independent trials. *P* < 0.05 was used as the threshold for determining statistical significance. GraphPad Prism 8 software was used to prepare figures and perform statistical comparisons.

## Supplementary Information

**Additional file 1: Figure S1.** Annexin V-propidium iodide flow cytometry plots of Jurkat cells treated with or without FSS for 1 h. Average cell viability of Jurkat cells treated with or without FSS (*N* = 3). Error bars are SD.

**Additional file 2: Figure S2.** Western blot of Piezo1 and GAPDH expression in Jurkat cells treated with Cas9 and sgRNA targeted to Piezo1, or Jurkat cells treated with Cas9 only (*N* = 1).

**Additional file 3: Figure S3.** Schematic of flow cytometry gating of T cells isolated from peripheral blood.

**Additional file 4.** Uncropped western blot gel of Piezo1 and GAPDH expression.

## Data Availability

Data and materials are available from the corresponding author upon reasonable request.

## Abbreviations

FSS: Fluid shear stress
ZAP70: Zeta-chain-associated protein kinase-70
NFAT: Nuclear factor of activated T cells
NF-κB: Nuclear factor kappa B
AP-1: Activator protein 1
TNF-α: Tumor necrosis factor alpha
IL-2: Interleukin 2
IFN-γ: Interferon gamma
EGTA: Ethylene glycol-bis(2-aminoethylether)-N,N,N',N'-tetraacetic acid
CCD: Cytochalasin D
CSA: Cyclosporin A
CAR: Chimeric antigen receptor
TRPV4: Transient receptor potential cation channel subfamily V member 4
FBS: Fetal bovine serum
BSA: Bovine serum albumin
DMSO: Dimethyl sulfoxide
HBSS: Hank’s balanced salt solution
GAPDH: Glyceraldehyde-3-phosphate dehydrogenase
G: Shear rate
*ω*: Angular velocity (rad/s)
*θ*: Angle of the cone (rad)
*τ*: FSS (dyn/cm^2^)
*μ*: Viscosity (cP)
SD: Standard deviation
