# Supplementary figures and images for: Fluid shear stress enhances T cell activation through Piezo1
Source: BMC Biol. 2022 Mar 9;20:61. doi: 10.1186/s12915-022-01266-7 (PMC8904069; doi:10.1186/s12915-022-01266-7)

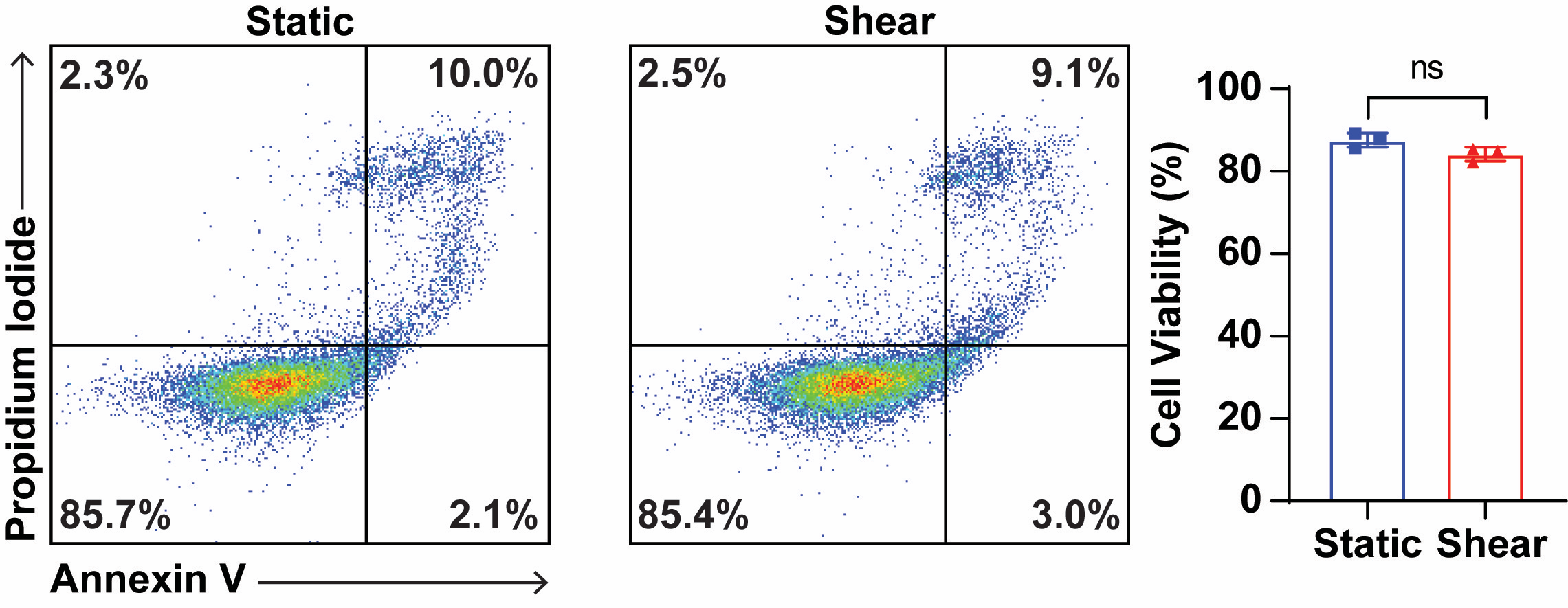

Supplement: Supplementary file 1 — Additional file 1: Figure S1. Annexin V-propidium iodide flow cytometry plots of Jurkat cells treated with or without FSS for 1 h. Average cell viability of Jurkat cells treated with or without FSS (N = 3). Error bars are SD. [file 12915_2022_1266_MOESM1_ESM.png]

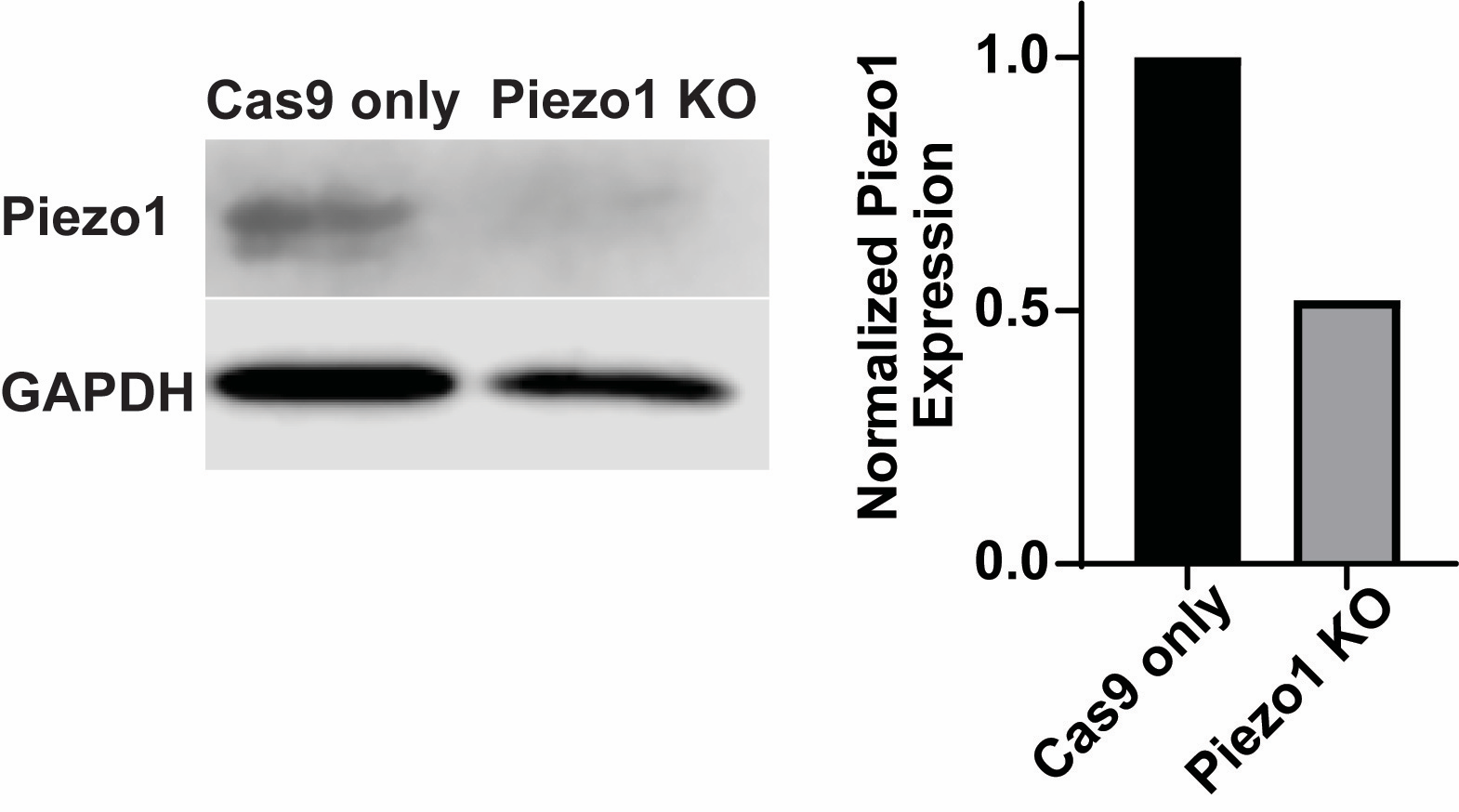

Supplement: Supplementary file 2 — Additional file 2: Figure S2. Western blot of Piezo1 and GAPDH expression in Jurkat cells treated with Cas9 and sgRNA targeted to Piezo1, or Jurkat cells treated with Cas9 only (N = 1). [file 12915_2022_1266_MOESM2_ESM.png]

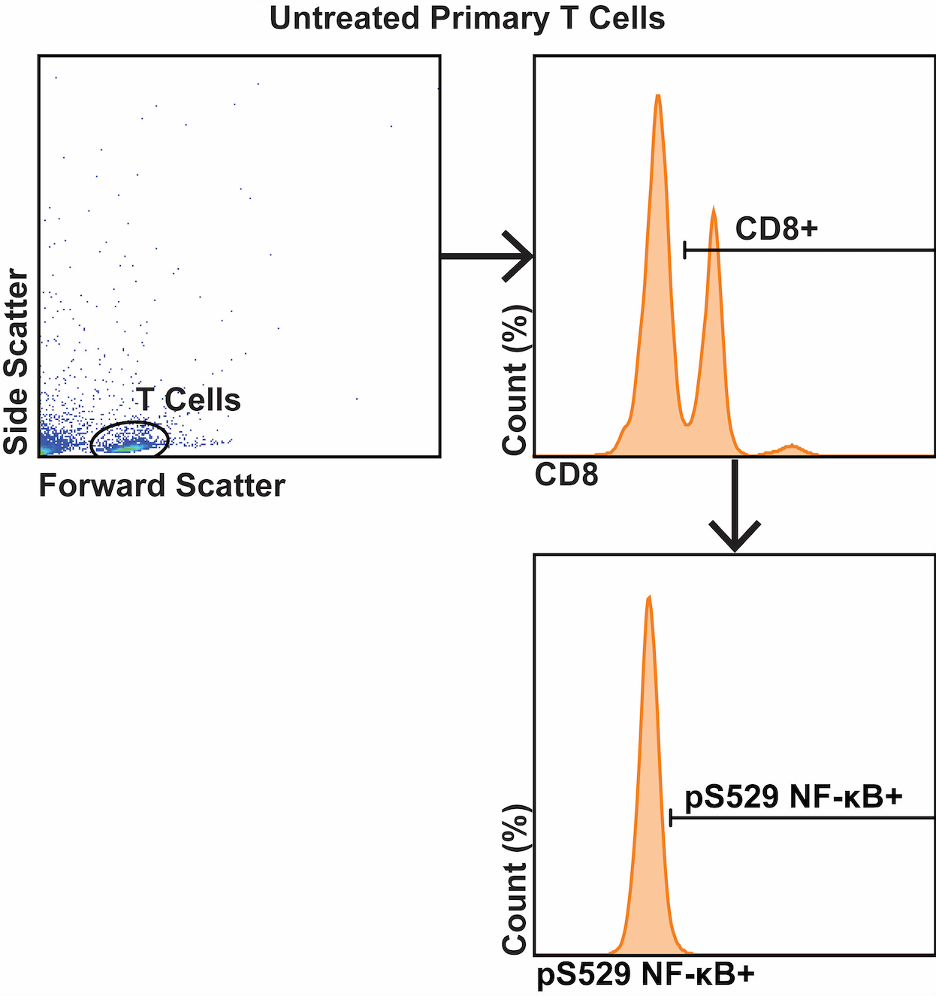

Supplement: Supplementary file 3 — Additional file 3: Figure S3. Schematic of flow cytometry gating of T cells isolated from peripheral blood. [file 12915_2022_1266_MOESM3_ESM.png]

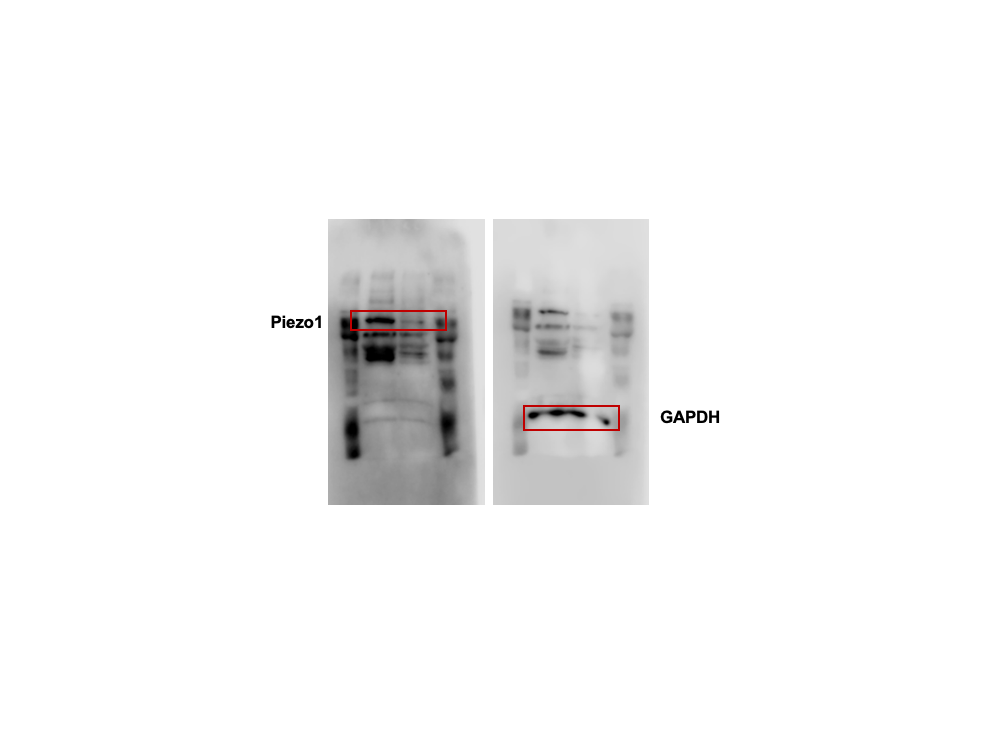

Supplement: Supplementary file 4 — Additional file 4. Uncropped western blot gel of Piezo1 and GAPDH expression. [file 12915_2022_1266_MOESM4_ESM.png]
